# Bivariate genetic association analysis of systolic and diastolic blood pressure by copula models

**DOI:** 10.1186/1753-6561-8-S1-S72

**Published:** 2014-06-17

**Authors:** Stefan Konigorski, Yildiz E Yilmaz, Shelley B Bull

**Affiliations:** 1 Dalla Lana School of Public Health, University of Toronto, 155 College Street, Toronto, ON, M5T 3M7, Canada; 2 Current address: Molecular Epidemiology Group, Max Delbrück Center for Molecular Medicine (MDC), Robert-Rössle-Straße 10, 13125 Berlin, Germany; 3 Lunenfeld-Tanenbaum Research Institute of Mount Sinai Hospital, 60 Murray Street, Box 18, Toronto, ON, M5T 3L9, Canada; 4 Current address: Department of Mathematics and Statistics, Memorial University of Newfoundland, St. John's, NL, A1C 5S7, Canada

## Abstract

We conduct genetic association analysis in the subset of unrelated individuals from the San Antonio Family Studies pedigrees, applying a two-stage approach to take account of the dependence between systolic and diastolic blood pressure (SBP and DBP). In the first stage, we adjust blood pressure for the effects of age, sex, smoking, and use of antihypertensive medication based on a novel modification of censored regression. In the second stage, we model the bivariate distribution of the adjusted SBP and DBP phenotypes by a copula function with interpretable SBP-DBP correlation parameters. This allows us to identify genetic variants associated with each of the adjusted blood pressures, as well as variants that explain the association between the two phenotypes. Within this framework, we define a pleiotropic variant as one that reduces the SBP-DBP correlation. Our results for whole genome sequence variants in the gene *ULK4 *on chromosome 3 suggest that inference obtained from a copula model can be more informative than findings from the SBP-specific and DBP-specific univariate models alone.

## Background

A number of genome-wide association studies (GWAS) involving large populations have been conducted to identify genetic variants associated with various single blood pressure (BP) measures: systolic blood pressure (SBP), diastolic blood pressure (DBP), or a linear function of them. Although the correlation between SBP and DBP is high, the results of the GWAS for each separately indicate only partially overlapping sets of variants associated with SBP and DBP. In this report, we model SBP and DBP jointly, taking the association between them into account. Constructing a bivariate model for these two phenotypes can increase the power to detect causal variants for one or both phenotypes, shedding more light onto the complex underlying genetic processes.

We apply copula functions [1] to model the bivariate distribution of SBP and DBP conditional on genetic variants. Copulas are functions used to construct a joint distribution by combining the marginal distributions with a dependence structure, and they allow investigation of the dependence structure between the phenotypes SBP and DBP separately from the marginal distributions. This property of copula models is very useful in identifying genetic variants that explain the dependence between SBP and DBP. It is well known that the Pearson correlation coefficient effectively measures the linear dependence of two random variables coming from a bivariate normal distribution. However, it may not be a good measure for other bivariate distributions where the conditional mean of $Y_{i}$ given $Y_{j}$ is not linear in $Y_{j}$. Hence, we prefer a nonparametric correlation measure. One frequently used measure based on concordance and discordance is Kendall's tau, which is the probability of concordance minus the probability of discordance. We also use upper and lower tail dependence measures, which measure the level of dependence in the upper-right quadrant tail and lower-left quadrant tail of a bivariate distribution, respectively, as it might be especially interesting to find pleiotropic variants explaining association between high SBP and high DBP or low SBP and low DBP.

Our analysis has two objectives: (a) to investigate the association of some common variants with SBP and DBP under the joint model of SBP and DBP, and (b) to identify pleiotropic variants, which we define as variants that explain the association between SBP and DBP.

## Methods

### San Antonio family studies data

The Genetic Analysis Workshop 18 (GAW18) data set includes 153 unrelated individuals from the San Antonio family studies pedigrees having SBP and DBP measurements at one or more study exams, information regarding current use of antihypertensive medication, and nongenetic covariates sex, age, and current tobacco smoking status at some examinations. To retain all subjects, we imputed missing age values by adding the mean time interval between measurements to the last known age of a subject. Some missing values of smoking status were also imputed by examining the smoking patterns of individuals over the four time points. We later verified that these imputations did not lead to any significant differences in parameter estimates and inference. Among the 153 unrelated individuals with measured phenotype data, 100 have whole genome sequence data. We conducted our genetic analysis on chromosome 3 in this group, considering only the *ULK4 *gene previously found to be associated with DBP [2]. We analyzed 1771 variants with minor allele frequency (MAF) ≥0.05.

### Phenotype definition

Before modeling the joint distribution of SBP and DBP conditional on genetic variants, we first adjusted the observed BPs for the effect of antihypertensive medication and other nongenetic covariates. The GAW18 unrelated pedigree members include hypertensive individuals (ie, with high BP) and some taking antihypertensive medication (Table 1). Adjusting BP for the effect of BP-lowering medication is crucial when the objective is to identify genes associated with high or low BP. Based on a simulation study comparing several methods [3], the use of a censored regression model conditional on both nongenetic and genetic covariates was recommended, assuming that a treated individual's true "underlying" BP is higher than that observed. For our analysis, we extend their censored regression approach by deriving the maximum likelihood estimate (MLE) of a conditional expectation that is in the form of fitted BP plus a nonnegative adjustment term depending on the observed BP and the nongenetic covariates. It provides a more intuitive adjustment of the BP of treated individuals than, for example, the nonparametric method or assuming that treatment has the same constant effect for each individual as in Tobin et al [3].

**Table 1:** Number of unrelated individuals, hypertensive individuals, and individuals on antihypertensive medication

	Exam 1	Exam 2	Exam 3	Exam 4
Total*	141	97	98	37
Hypertensive†	42	49	52	27
Receiving meds	27	30	45	25

*Number of unrelated individuals having SBP and DBP measurements and current use of antihypertensive medication available at the examination time point used in the phenotype adjustment.

†Number of hypertensive individuals (SBP >140 mm Hg or DBP >90 mm Hg or on antihypertensive medications at that examination).

At each examination point *j*(j=1,2,3,4), we separately fitted censored regression models of BP conditional on nongenetic covariates age, sex, and smoking status with medication use as the censoring indicator. After conducting standard residual analysis and model selection, we specified the models as

(1) $$SBP_{i,j}=\gamma_{0\left(j\right)}+\gamma_{1\left(j\right)}\;sex_{i}+\gamma_{2\left(j\right)}\;smoke_{i,j}+\gamma_{3\left(j\right)}\left(age_{i,j}-\bar{age_{j}}\right)+\epsilon_{i,j}$$

(2) $$DBP_{i,j}={\gamma_{0\left(j\right)}}^{\prime }+{\gamma_{1\left(j\right)}}^{\prime }\;sex_{i}+{\gamma_{2\left(j\right)}}^{\prime }\;smoke_{i,j}+{\gamma_{3\left(j\right)}}^{\prime }|age_{i,j}-{\bar{age}}_{j}|+{\epsilon_{i,j}}^{\prime }$$

where $\epsilon_{i,j}\sim N\left(0,{\sigma_{SBP,j}}^{2}\right)$, ${\epsilon_{i,j}}^{\prime }\sim N\left(0,{\sigma_{DBP,j}}^{2}\right)$, $\bar{age_{j}}=\frac{1}{n_{j}}\overset{n_{j}}{\underset{i=1}{\sum }}age_{i,j}$, and $i=1,\ldots ,n_{j}$$(n_{1}=141,n_{2}=97,n_{3}=98,n_{4}=37)$. This formulation of the age covariates reflects previous findings (see, eg, Ref. [4]) that SBP increases with age whereas DBP decreases after the age of 55 to 60 years, which can be approximated here with the sample mean age. We used the "survreg" function in the "survival" package of R to fit the censored regression models.

**Figure 1:**
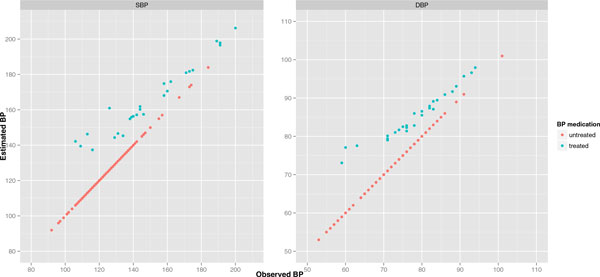
**Scatterplots of observed versus adjusted BP for the first examination time, stratified by SBP and DBP**. For untreated individuals, adjusted BPs are equal to the observed BPs; consequently, red points fall on the diagonal.

For individuals who received antihypertensive medication, we estimate the underlying BP with the MLE of the conditional expectation of BP, given that the observed BP is lower than the true underlying BP. For illustration, under the model (1), the conditional expectation is

(3) $$E\left[SBP_{i,j}|SBP_{i,j}>SBP_{obs,i,j},Z_{i,j}=z_{i,j}\right]=\gamma_{\left(j\right)}z_{i,j}+\frac{{\sigma_{SBP,j}}^{2}f\left(SBP_{obs,i,j}|z_{i,j}\right)}{1-F\left(SBP_{obs,i,j}|z_{i,j}\right)}$$

where $z_{i,j}$ denotes the vector of nongenetic covariates with associated regression parameter $\gamma_{\left(j\right)}=\left(\gamma_{0\left(j\right)},\gamma_{1\left(j\right)},\gamma_{2\left(j\right)},\gamma_{3\left(j\right)}\right),$ and f,F are the normal probability density and cumulative distribution functions, respectively, with mean $\gamma_{\left(j\right)}z_{i,j}$ and variance ${\sigma_{SBP,j}}^{2}$. The effects of the adjustment are evident in Figure 1, with the adjusted BP of treated individuals always higher than their observed BP. At each of the first 3 examination time points, we obtained residuals from fitting the censored regression models (1) and (2). We disregarded the last examination time because few BP measurements were available (see Table 1). An untreated individual's residual is the difference between observed and fitted BPs; a treated individual's residual is the difference between adjusted and fitted BP. Finally, we averaged the residuals (over *j *= 1,2,3) separately for SBP and DBP, and took these mean residuals as our adjusted phenotypes.

### Bivariate copula modeling

In the second stage, we first constructed the marginal models for our adjusted phenotypes $Y_{1}$ and $Y_{2}$ given a genetic variant X=x, assuming that the genetic variants *X *are independent of the nongenetic variants *Z*. In the marginal models

(4) $$Y_{1,i}=\alpha_{0}+\alpha_{1}x_{i}+\epsilon_{i}\;\;\text{and}\;\;Y_{2,i}=\beta_{0}+\beta_{1}x_{i}+{\epsilon_{i}}^{\prime }$$

we observed no evidence against the normality assumptions for the error terms. We then used a copula function *C *to build the bivariate distribution of $Y_{1}$ and $Y_{2}$ conditional on genetic variants by combining the 2 marginal distribution functions $F_{1}\left(y_{1}\text{|}X=x\right)$ and $F_{2}\left(y_{2}\text{|}X=x\right)$. More specifically, we consider the bivariate distribution function

(5) $$F\left(y_{1},y_{2}\text{|}X=x\right)=C_{\psi }\left(F_{1}\left(y_{1}\text{|}X=x\right),F_{2}\left(y_{2}\text{|}X=x\right)\right)$$

where $F_{1}$ and $F_{2}$ are the normal cumulative distribution functions with variances ${\sigma_{1}}^{2}$ and ${\sigma_{2}}^{2}$, respectively, and *ψ *is the vector of copula parameters. To illustrate the approach, consider the 2-parameter copula family

(6) $$C_{\psi }\left(u_{1},u_{2}\right)={\left\{{\left[{\left({u_{1}}^{-\varphi }-1\right)}^{\theta }+{\left({u_{2}}^{-\varphi }-1\right)}^{\theta }\right]}^{1/\theta }+1\right\}}^{-1/\varphi }$$

with $0\leq u_{1},u_{2}\leq 1,$ and the copula (or dependence) parameters $\psi =\left(\varphi ,\theta \right),\varphi >0,\theta \geq 1$. To explain the association between $Y_{1}$ and $Y_{2}$, we use Kendall's tau (τ), which is a measure of overall association based on concordance and discordance, and we use lower and upper tail dependence measures (*λ_L_*, *λ_U_*, respectively), which explain the amount of dependence between extreme values, and can give more insight in identifying pleiotropic variants. For the copula family in (6), these dependence measures become [1]

(7) $$\tau =1-\frac{2}{\theta \left(\varphi +2\right)},\;\lambda_{L}=2^{-1\;/\;\theta \varphi },\;\lambda_{U}=2-2^{1\;/\;\theta }$$

We obtain MLEs of the marginal parameters $\alpha =\left(\alpha_{0},\alpha_{1},\sigma_{1}\right)$, $\beta =\left(\beta_{0},\beta_{1},\sigma_{2}\right)$ in equation (4) and the copula parameters $\psi =\left(\varphi ,\theta \right)$ in equation (6) by maximizing the likelihood function [1] with the general optimization software implemented in the nlm function in R. Variance estimates for the MLEs are obtained from the inverse of the observed information matrix.

To address aim (a) concerning the marginal association of a variant with each SBP and DBP under the bivariate model (5), we test the null hypotheses $H_{0}:\alpha_{1}=0$ (vs. $H_{A}:\alpha_{1}\neq 0$) and $H_{0}:\beta_{1}=0$ (vs. $H_{A}:\beta_{1}\neq 0$) with the large sample Wald test statistic. We expect improved inference under the bivariate model compared to inference obtained by separate analysis of SBP and DBP, which we refer to as the working independence model.

In contrast, for aim (b), which is to identify a variant that explains association between SBP and DBP, the copula model dependence parameters *φ *and *θ*, and dependence measures (7) are of interest. We compare estimates of Kendall's $\tau \;\lambda_{L}$ and $\lambda_{U}$ under the full bivariate model (5) that includes the genetic variant with the corresponding estimates obtained under the bivariate model without the variant (ie, the null model with $H_{0}:\alpha_{1}=\beta_{1}=0$). According to the delta method, we construct a confidence interval (CI) for the dependence measures using large-sample standard errors. When the CIs for a given association measure under the null and the full model do not overlap, we conclude that the given variant is pleiotropic. Use of CIs in this way is quite conservative. We also check whether the CI for $\lambda_{L}$ or $\lambda_{U}$ under the full model includes 0. Note that the copula model (6) only becomes an independent copula $C\left(u_{1},u_{2}\right)=u_{1}u_{2}$ when θ=1 and *φ *goes to 0. However, because it is practically impossible to identify all variants, instead of testing independence, we search for variants that reduce the magnitude of the overall dependence measures, such as Kendall's *τ*.

## Results and discussion

For model selection, we note that the Akaike information criterion (AIC) value under the copula model (6) is much lower than the AIC under a bivariate normal model, indicating that the copula model is a better fit. For example, the AIC value under the copula model (6) reported in Table 2 is 1227.6 compared to an AIC value of 1337.8 under the bivariate normal model (not shown). These AICs are comparable to those obtained when conditioning on other variants. The aim (a) results (Table 2) are thus limited to the Wald test *p *values of the MLE estimates of the coefficients $\alpha_{1}$, $\beta_{1}$ in (4) for testing $H_{0}:\alpha_{1}=0$ and $H_{0}:\beta_{1}=0$ under two models: the working independence model and the bivariate copula model (6) for single-variant analysis. We observed some variants, including less common (0.05 ≤ MAF ≤0.10) and more common (MAF >0.10) variants, that are identified by both models, but the *p *values for testing $H_{0}:\alpha_{1}=0$ and $H_{0}:\beta_{1}=0$ under the copula model (with minimum *p *values 1.7 × 10^−4 ^and 5.1 × 10^−5^, respectively) are smaller than the *p *values under the working independence model (with minimum *p *values 5.5 × 10^−3 ^and 7.0 × 10^−4^, respectively). This includes variants significantly associated with both BP phenotypes under the joint copula model, although they are not significantly associated at the 1% level with either under the univariate phenotypic models (see Table 2). Overall, the estimated genetic effect sizes are larger and the estimated standard errors are slightly smaller under the bivariate model.

**Table 2:** Results of testing ${\text{H}}_{0}:\alpha_{1}=0$ or ${\text{H}}_{0}:\beta_{1}=0$ for variant at 41,984,243 base-pair position

	SBP		DBP	
		
	Working independence	Bivariate copula	Working independence	Bivariate copula
Coefficient Estimate (SE)	11.1 (5.3)	16.7 (4.7)	7.2 (2.9)	8.4 (2.7)
*p *Value	4.0 × 10^−2^	3.8 × 10^−4^	1.5 × 10^−2^	1.6 × 10^−3^

The AIC value under the working independence model of SBP and DBP is 1318.4, and the AIC value under the bivariate copula model (6) is 1227.6.

**Figure 2:**
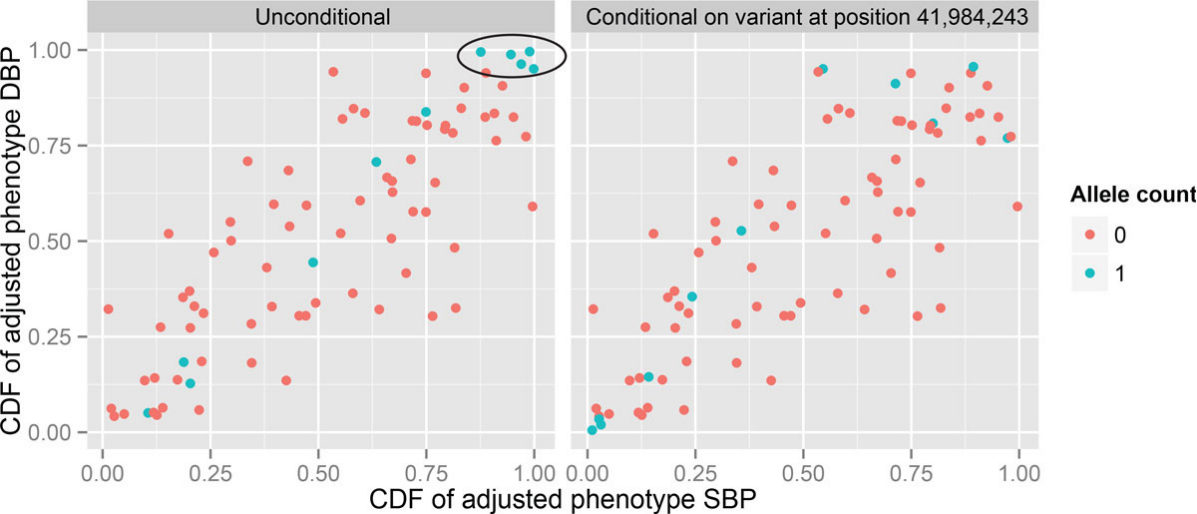
Scatterplots of the cumulative distribution function of $Y_{1}\left(F_{1}\right)$ versus $Y_{2}\left(F_{2}\right)$ without conditioning on any variant (*left panel*) and conditional on the variant at 41,984,243 base-pair position (*right panel*). The indicated blue dots denote individuals with high adjusted phenotypes having 1 minor allele of the variant (no individual has 2 alleles). Without conditioning on the variant, these dots in the upper tail are closely clustered (*left panel*), but when the effect of the variant is removed (*right panel*), they spread out. Note that the unexplained association is likely to be caused by other unknown variants. If we could have identified all the variants that explain the association between phenotypes, then the plot in the right panel conditioning on these variants would have all dots randomly scattered on the unit square.

Table 3 displays 2 of 10 variants yielding a substantial reduction in point estimates of the upper tail dependence measure $\lambda_{U}$ under the bivariate model (5) conditional on the variant. Compared to the null model when conditioning on a variant in the gene *ULK4*, Kendall's tau and lower tail dependence do not differ markedly. We observe that without conditioning on any variant $\left(H_{0}:\alpha_{1}=\beta_{1}=0\right)$, the 2 phenotypes are moderately correlated with a Kendall's tau estimate of 0.578, and higher lower tail dependence than upper tail dependence (Table 3). Conditioning on the variant at 41,984,243 base-pair position diminishes upper tail dependence measure $\lambda_{U}$ at 0.01 level of significance (99% CI for $\lambda_{U}$ includes 0); this variant is also associated with SBP and DBP (see Table 2). Figure 2 illustrates how it achieves a reduction in upper tail dependence. Tail dependence can also be reduced in the absence of strong marginal BP associations. For example, the variant at 41,971,559 base-pair position is only modestly associated with SBP (*p *value = 0.040) and DBP (*p *value = 0.055), but the upper tail dependence is reduced from 0.449 obtained under the null model to 0.289 with a CI that includes 0 (Table 3).

**Table 3:** Estimates of dependence measures $\left(\tau ,\lambda_{L},\lambda_{u}\right)$ under the null model $H_{0}:\alpha_{1}=\beta_{1}=0$ and under model (5) in a single-variant analysis

Variant	$\hat{\tau }$ (SE)	(99% CI for τ)	$\hat{\lambda_{L}}$ (SE)	(99% CI for $\lambda_{L}$)	$\hat{\lambda_{U}}$ (SE)	(99% CI for $\lambda_{u}$)
Null	0.578 (0.05)	(0.449, 0.707)	0.650 (0.07)	(0.470, 0.830)	0.449 (0.10)	(0.191, 0.707)
41,984,243	0.555 (0.06)	(0.400, 0.710)	0.703 (0.06)	(0.548, 0.858)	0.268 (0.21)	(0.000, 0.809)
41,971,559	0.570 (0.05)	(0.441, 0.699)	0.715 (0.06)	(0.560, 0.870)	0.289 (0.18)	(0.000, 0.753)

## Conclusions

In this report, we demonstrate how to model the bivariate distribution of SBP and DBP with copulas and conduct appropriate inference for genetic association. The proposed method is shown to be applicable by considering a single gene, and crude estimates of computation time suggest that it is feasible to process 1 million variants in less than a day, for example, by using one hundred 2.5-GHz cores. Although estimating the bivariate distribution is computationally more intensive than fitting the working independence model, given the high correlation between the phenotypes, a potential advantage is that genetic associations can be detected with higher power under a plausible joint model. Using joint copula models, we were also able to identify candidate variants explaining the upper tail dependence of SBP and DBP. We generally observed strong linkage disequilibrium between variants identified. By conducting joint analyses of multiple variants in moderate linkage disequilibrium, we achieved a much more significant reduction in upper tail dependence (data not shown), and although we observed some reduction in point estimates of lower tail dependence and Kendall's tau, the CIs still overlap with those under the null model. Calling a comparison significant when the CIs fail to overlap is a conservative approach, but it is computationally efficient. As an alternative, a nonparametric bootstrap procedure could be used to estimate the variance of the estimated difference between dependence measures under the null and full models, and to construct an approximate CI. To allow multiple testing adjustments, instead of checking whether the CI for $\lambda_{L}$ or $\lambda_{U}$ under the full model includes 0, it would be desirable to test each of the null hypotheses $H_{0}:\varphi =0$ or $H_{0}:\theta =1$, respectively, to obtain *p *values [5].

In principle, the extension of our approach to 3 or more quantitative traits is straightforward; however, the copula model (6) may not be ideal in this setting. It involves some restrictions on the association structure, and generally the Gaussian copula is used when there are 3 or more traits. The approach could also be extended to binary traits, but with some caution because there is no unique copula identifying the joint distribution function of discrete variables [6].

## Competing interests

The authors declare that they have no competing interests.

## Authors' contributions

YEY and SBB designed the overall study, SK conducted statistical analyses, and SK and YEY drafted the manuscript. All authors read and approved the final manuscript.
